# Validating surgical procedure codes for inflammatory bowel disease in the Swedish National Patient Register

**DOI:** 10.1186/s12911-019-0948-z

**Published:** 2019-11-11

**Authors:** Anders Forss, Pär Myrelid, Ola Olén, Åsa H. Everhov, Caroline Nordenvall, Jonas Halfvarson, Jonas F. Ludvigsson

**Affiliations:** 1 Department of Medical Epidemiology and Biostatistics, Karolinska Institutet, Box 281, SE-171 77 Stockholm, Sweden; 2 Department of Clinical and Experimental Medicine, Faculty of Health, Linköping University, Department of Surgery, Linköping University Hospital, Linköping, Sweden; 3 Clinical Epidemiology Unit, Department of Medicine Solna, Karolinska Institutet, Stockholm, Sweden; 4 Sachs’ Children and Youth Hospital, Stockholm South General Hospital, Stockholm, Sweden; 5 Department of Clinical Science and Education, South General Hospital, Stockholm, Sweden; 6 Department of Molecular Medicine and Surgery, Karolinska Institutet, Stockholm, Sweden; 7 Center for Digestive Disease, Division of Coloproctology, Karolinska University Hospital, Stockholm, Sweden; 8 Department of Gastroenterology, Faculty of Medicine and Health, Örebro University, Örebro, Sweden; 9 Department of Paediatrics, Örebro University Hospital, Örebro, Sweden; 10 Division of Epidemiology and Public Health, School of Medicine, University of Nottingham, Nottingham, UK; 11 Department of Medicine, Columbia University College of Physicians and Surgeons, New York, New York, USA

**Keywords:** Epidemiology, Inflammatory bowel disease, Validation, National Patient Register, Procedure code

## Abstract

**Background:**

About 50% of patients with Crohn’s disease (CD) and about 20% of those with ulcerative colitis (UC) undergo surgery at some point during the course of the disease. The diagnostic validity of the Swedish National Patient Register (NPR) has previously been shown to be high for inflammatory bowel disease (IBD), but there are little data on the validity of IBD-related surgical procedure codes.

**Methods:**

Using patient chart data as the gold standard, surgical procedure codes registered between 1966 and 2014 in the NPR were abstracted and validated in 262 randomly selected patients with a medical diagnosis of IBD. Of these, 53 patients had reliable data about IBD-related surgery. The positive predictive value (PPV), sensitivity and specificity of the surgical procedure codes were calculated.

**Results:**

In total, 158 surgical procedure codes were registered in the NPR. One hundred fifty-five of these, representing 60 different procedure codes, were also present in the patient charts and validated using a standardized form. Of the validated codes 153/155 were concordant with the patient charts, corresponding to a PPV of 96.8% (95%CI = 93.9–99.1). Stratified in abdominal, perianal and other surgery, the corresponding PPVs were 94.1% (95%CI = 88.7–98.6), 100% (95%CI = 100–100) and 98.1% (95%CI = 93.1–100), respectively. Of 164 surgical procedure codes in the validated patient charts, 155 were registered in the NPR, corresponding to a sensitivity of the surgical procedure codes of 94.5% (95%CI = 89.6–99.3). The specificity of the NPR was 98.5% (95%CI = 97.6–100).

**Conclusions:**

Data on IBD-related surgical procedure codes are reliable, with the Swedish National Patient Register showing a high sensitivity and specificity for such surgery.

## Background

The prevalence of inflammatory bowel disease (IBD) in Sweden is estimated at 0.65% [1] with a mean incidence rate of 37/100,000 person-years [2]. Treatment of IBD stands on three pillars: medical treatment, nutritional support and surgery. While pharmacological treatment is the mainstay for IBD, studies indicate that about 50% of patients with Crohn’s disease (CD) and about 20% of those with ulcerative colitis (UC) undergo surgery sometime during their lifetime [2–8]. Surgery is rarely curative in CD, whereas colectomy can radically reduce the disease burden in UC, improving short- and long-term quality of life [9, 10]. Surgery is also elemental in the treatment of colorectal cancer, a feared complication of long-standing colonic IBD [11]. In Sweden, most diagnoses and procedure codes used for register-based studies are identified through the Swedish National Patient Register (NPR) [12]. The NPR, formed in 1964, is a nationwide register containing data on discharge diagnoses and procedure codes of all patients admitted to hospital. The register became nationwide in 1987. Data on hospital-based outpatient care were added in 2001, lending the NPR a potential coverage of nearly 100% [10]. At the time of forming the NPR, a new standard for classification of surgical procedure codes was introduced. In 1997, it was replaced by an adapted version of the NOMESCO Classification of Surgical Procedures [13] and registration of day surgery was included. The procedure codes have been revised by the Swedish Board of Health and Welfare and listed in the publication Swedish Classification of Surgical and Medical Procedures (in Swedish: “KVÅ” – klassifikation av. vårdåtgärder) [14]. The NPR and quality registers in Sweden provide unique opportunities for systematic collection of medical data at the population and individual level [15]. Through the NPR, health care personnel and researchers can determine the incidence and prevalence of IBD. Based on the procedure codes related to IBD surgery in the NPR the effects and consequences of surgical interventions in IBD patients can be studied, both in large epidemiological and in smaller clinical studies. To ensure high validity and reliability of such future research, IBD related surgical procedure codes needs to be validated. The procedure codes validated in this study include codes also used for similar surgical interventions in other diseases than IBD, such as colorectal surgery in cancer patients. We have previously validated the medical diagnoses of IBD [16]. Meanwhile, studies validating surgical procedure codes in the NPR are scarce; however, positive predictive values (PPVs) of 99.6% for oesophageal surgery [17] and 97.0% for obesity surgery [18] have been shown. To our knowledge, no study has assessed the validity of IBD-related surgical procedure codes or the sensitivity for those codes in the NPR. The present study aimed to validate IBD-related surgical procedure codes assessing the PPV, sensitivity and specificity for those codes in the NPR.

## Methods

This study was a structured retrospective review comparing IBD-related surgical procedure codes in the NPR with data from patient charts as the gold standard. The study took place in Sweden and review of the patient charts was done between June 2017 and November 2017.

### Study population

**Fig. 1:**
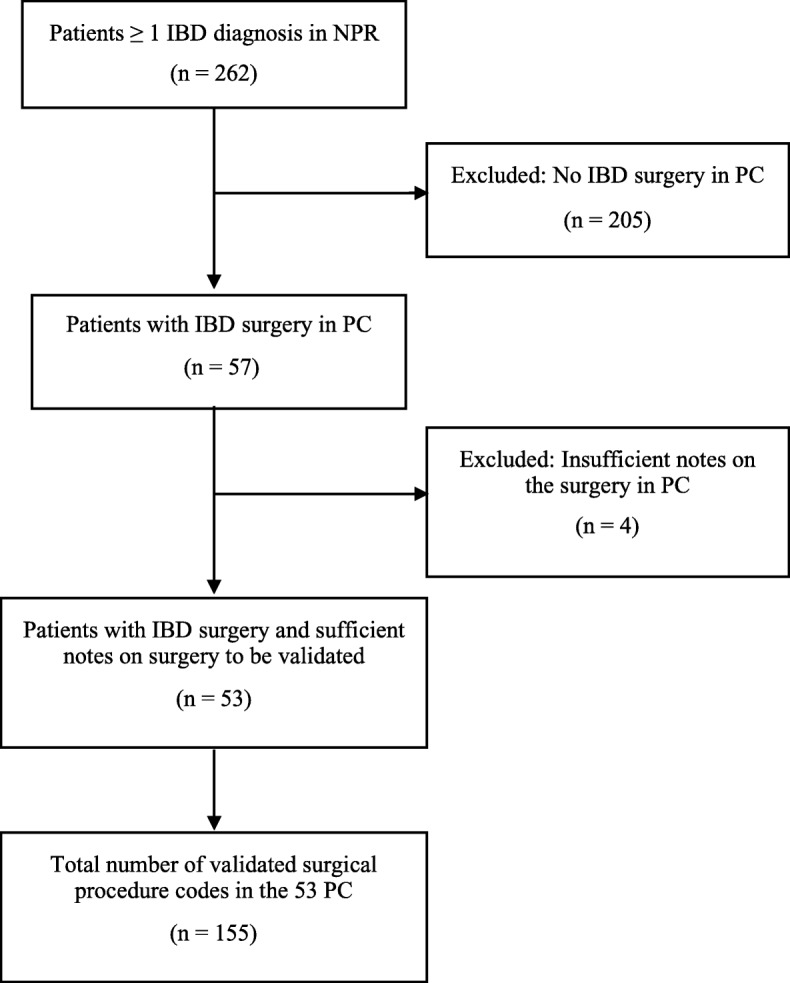
Flowchart for inclusion of the validation *IBD = inflammatory bowel disease, NPR = national patient register, PC = patient chart*

The study sample was extracted from cases of a previous study validating IBD diagnoses in the NPR [16]. These cases (*n* = 370) were randomly identified by the Swedish Board of Health and Welfare, meeting the inclusion criterion of at least one diagnosis of IBD (ICD-9: CD 555, UC 556 or ICD-10: CD K50, UC K51) registered in the NPR between 1987 and 2014 [16]. Retrieved data from the NPR included the patients’ personal identity number (PIN), hospital, department, date of IBD diagnosis (index date) and surgical procedures. Hospitals and departments having treated the patient were asked to provide all available physicians or surgery notes, discharge summaries, laboratory test results and radiology/histopathology/endoscopy referrals 2 years before and at least 2 years after the index date defined as date of first diagnosis of IBD between 1987 and 2014 [16]. However, several of the hospitals submitted patient chart information preceding the requested period, for some patients as far back as 1966. Such data were also included in the validation. In total, 293/370 requested charts were received. Of these, 262 were physically available for this study. The remaining 31 were only accessible in a database to which the main investigator of this study (AF) was not authorised to access. The charts of 57 patients contained information on IBD-related surgery between 1966 and 2014, whereas the remaining 205 patient charts did not. Four patients were excluded because of insufficient notes about surgery, i.e. the patient charts contained insufficient information to determine type of surgery undertaken and therefore did not allow any reliable validation. Thus, the final study population consisted of 53 patients, an overview of the selection process of patient charts included in this study is described in Fig. 1.

### Data elements

#### Extraction of data

**Fig. 2:**
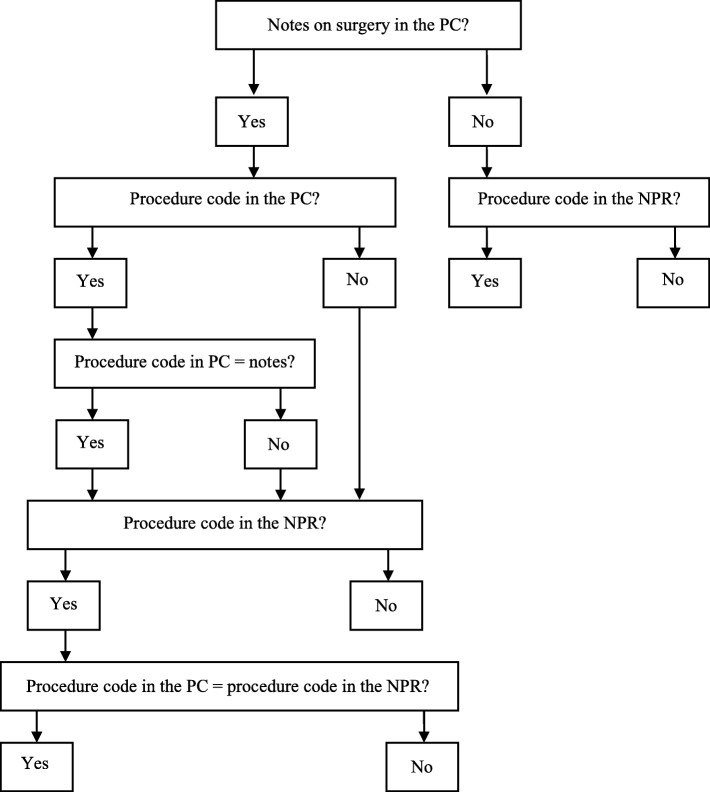
Flowchart of review form for IBD-related surgical procedure codes *NPR = national patient register, PC = patient chart*

Data from the patient charts on IBD-related surgery were abstracted together with existing surgical procedure codes classified as IBD-related (Additional file 1: Table S1) using a standardised review form (Fig. 2). Abstracted information included date of surgery, surgical procedure code or description of the surgery in surgical or other notes.

#### Review process of patient charts

The patient charts were reviewed by a single reviewer (AF). The abstracted surgical procedure codes in the charts were compared with the surgical notes, which served as the gold standard to determine which surgical procedure the patient had undergone. If such notes were absent, other notes were used that clearly described the procedure undertaken. In case of ambiguous interpretation each case was discussed with an experienced IBD surgeon (PM). The abstracted data were then compared with the NPR to determine whether any procedure code was registered for the same surgical procedure and whether the code was concordant with the NPR. All abstracted data were reviewed twice before calculations.

### Case definition

The surgical procedure codes in the NPR were classified as “confirmed” (yes) if concordant with the information on the surgical procedures in the patient charts or “false” (no) if absent or if not concordant with the patient charts. If the code in the NPR were classified as “false”, the error was then categorised by type of error (Additional file 1: Table S2).

### Statistics

We expressed the concordance of surgical procedure codes in the NPR as a positive predictive value (PPV). The sensitivity for those codes in the NPR was expressed as the proportion of patient chart codes also present in the NPR and the specificity as the proportion of patient charts negative for IBD-related surgery also negative for such surgery in the NPR. We calculated 95% confidence intervals (CIs) for these accuracy measures using a two-step bootstrap approach clustered for hospital in strict hierarchy with 10,000 re-samplings [19, 20]. Data management was performed using Microsoft Excel, STATA software version 14.2 and R software version 3.4.1.

## Results

We reviewed patient charts from 262 patients identified as IBD patients in the NPR (Table 1). Surgical procedure codes for IBD surgery were registered in the NPR for 57 (22%) of these patients. The patient charts of these included information about IBD-related surgery, i.e. surgery notes, other notes or surgical procedure codes indicating surgery. After reviewing these charts, four patients were excluded because of insufficient information on type of surgery in the patient charts to allow validation (Fig. 1). The remaining 53 charts comprised data on 164 surgical procedures registered between 1966 and 2014 classified as IBD-related (Additional file 1: Table S1) in patients with a medical IBD diagnosis in the NPR registered at 27 different hospitals. Of these, nine (5%) codes were missing in the NPR (Table 2). The remaining 155 codes, representing 60 different surgical procedure codes, were validated using a standardized form (Fig. 2). In the NPR, 158 codes were registered for the included 258 patients. Of these, three (2%) codes were missing in the charts (Table 2). In total 153/155 validated surgical procedure codes were concordant, representing a PPV for true positive codes of 98.7% (95%CI = 96.3–100) and a PPV of 96.8% (95%CI = 93.9–99.1) for any IBD-related surgical procedure code in the NPR (*n* = 158) also being concordant. The two discrepancies between the codes in the charts and the NPR (one registered in 1988 and one in 2000) were both transfer errors (Additional file 1: Table S2), possibly technical translation errors occurring when manually transferring the procedure code from the patient chart to the register, for example JFB-- instead registered as JBF--. In a sensitivity analysis, including the four patients excluded from the main analysis due to insufficient information on type of surgery in the patient charts to allow validation, the PPV ranged between 96.2% (95%CI = 93.2–99.2) and 98.7% (95%CI = 97.0–100) for true positive codes and between 94.4% (95%CI = 90.9–98.0) and 96.9% (95%CI = 94.2–99.6) for any IBD-related code also being concordant. The validated codes were divided into categories according to the anatomic location of the procedure: abdominal (*n* = 65), perianal (*n* = 36) and other IBD locations (*n* = 54) (Table 3). When examining the validated codes stratified into these categories, the PPVs for any of them also being concordant were 94.1% (95%CI = 88.7–98.6) for abdominal, 100% (95%CI = 100–100) for perianal and 98.1% (95%CI = 93.1–100) for other IBD-related codes (Table 3). All perianal surgical procedure codes (n = 36) occurred in patients with a primary diagnosis of CD. Of the 155 validated surgery codes, 77 were registered up until 1996 and 78 in 1997 or later. Restricting the analyses to codes registered up until 1996 vs. 1997 or later, the corresponding PPVs were 97.4% (95%CI = 92.7–100) and 96.2% (95%CI = 92.9–100) (Table 3). Of 164 codes in the patient charts, 155 were found in the NPR corresponding to a sensitivity of 94.5% (95%CI = 89.6–99.3) (Table 2). Of these (seen in four patients), eight were registered in 1996 or earlier (Table 3) and consisted of six perianal procedure codes. One procedure code registered in 1997 or later was missing (Table 3). When restricted to up until 1996, the sensitivity was 90.6% (95%CI = 84.6–100) and 98.7% (95%CI = 95.0–100) in 1997 or later (Table 3). When including the four patients excluded from the main analysis due to insufficient information on type of surgery in the patient charts we found a sensitivity between 92.2% (95%CI = 88.2–96.3) and 94.6% (95%CI = 91.2–98.1). Out of the remaining 205 patients without an IBD-related surgical procedure code in the patient charts, 202 had no surgical procedure codes registered and three patients had one surgical procedure code each registered in the NPR. The overall specificity for those patients was calculated to 98.5% (95%CI = 97.6–100) (Table 2). We found 9 surgical procedure codes in the patient charts missing in the NPR (false negative codes). These codes were distributed between 4 different patients out of which 2 also had at least one additional surgical procedure code correctly registered in the NPR for the same surgical session (true positive codes). These 2 patients were both true positives and false negatives. However, through sensitivity analyses, we calculated the NPV conditioned all 4 patients or 2 patients as false negatives showing NPVs of 98.1% (95%CI = 96.2–100) and 99.0% (95%CI = 97.7–100) respectively. We calculated the number of days between admission registered in the NPR and the actual date of surgery according to the patient chart. For 90% of the patients (*n* = 76/84), surgery took place within 7 days (1 week) of the admission date. IBD-related surgery took place at a median of 1 (IQR 1.5) day and on average 2.1 (SD 3.1) days after the admission date.

**Table 1:** Basic characteristics of 262 patients in the Swedish National Patient Register included in the review

No. of reviewed patients	262
No. females/males (%)	119 (45)/143 (55)
Mean age (range) at first validated procedure code	38 (14–74)
No. of hospitals included	27
Mean patient chart coverage (years)	5.4
No. of patients with surgical procedures validated	53
No. of separate surgical occasions validated	103
No. of surgical procedure codes validated	155
Mean no. of surgical procedure codes (SD) per hospital	6.1 (13.2)
Mean no. of surgical procedure codes (SD) per patient	3.1 (4.5)
Mean no. of surgical sessions (SD) per patient	1.9 (2.6)

*NPR* National patient register, *SD* Standard deviation

**Table 2:** Positive predictive value, sensitivity and specificity of the Swedish National Patient register for IBD-related surgical procedure codes (n=258 patients)

NPR	Chart review	
	Patient chart positive for IBD-related surgery (*n* = 53 patients; *N* = 164 codes)	Patient chart negative for IBD-related surgery (*n* = 205 patients)
Code for IBD-related surgery (Concordant code in NPR)	155 (153)	3
No corresponding code for IBD-related surgery	9	202
Patients with no code in the NPR	2	
*PPV (true positives, 95%CI)*	98.7 (96.3–100) 153/155	
*PPV (concordant codes, 95%CI)*	96.8 (93.9–99.1) 153/158	
*Sensitivity of the NPR (95%CI)*	94.5 (89.6–99.3) 155/164	
*Specificity of the NPR (95%CI)*		98.5 (97.6–100) 202/205

*CI* Confidence interval, *IBD* Inflammatory bowel disease, *NPR* National patient register, *PPV* Positive predictive value PPV of true positives calculated from surgical procedure codes in the NPR present in the charts (*n* = 155) and codes confirmed by chart review. (*n* = 153). PPV of concordant codes calculated from all surgical procedure codes in the NPR (*n* = 158) and codes confirmed by chart review (*n* = 153)

**Table 3:** Positive predictive value and sensitivity of IBD-related surgical procedure codes in the Swedish National Patient Register stratified by type of surgery and year

Codes	Abdominal	Perianal	Other IBD	1966–1996	1997–2014	*Total*
Code in chart	67	40	57	85	79	*164*
Code in chart but missing in NPR	2	4	3	8	1	*9*
Code in NPR	68	36	54	78	80	*158*
Code in NPR but missing in chart	3	0	0	1	2	*3*
Code in chart and NPR	65	36	54	77	78	*155*
Correct code in chart and NPR	64	36	53	76	77	*153*
Correct code in chart but false in NPR	1	0	1	1	1	*2*
*PPV (95%CI)*	94.1 (88.7–98.6) 64/68	100 (100–100) 36/36	98.1 (93.1–100) 53/54	97.4 (92.7–100) 76/78	96.2 (92.9–100) 77/80	
*Sensitivity (95%CI)*	97.0 (91.9–100) 65/67	90.0 (81.8–100) 36/40	94.7 (88.9–100) 54/57	90.6 (84.6–100) 77/85	98.7 (95.0–100) 78/79	

*CI* Confidence interval, *IBD* Inflammatory bowel disease, *NPR* National patient register, *PPV* Positive predictive value Codes in the patient charts but missing in the NPR registered in 1996 or earlier consisted of six perianal procedure codes (KVÅ, 4999 - Other operation on anus or perianal tissue) and two abdominal procedure codes (KVÅ, 4642 - Ileocaecal resection; KVÅ, 4611 - Laparatomy and closure of a fistula to the bowel wall). One code registered in 1997 or later was missing (NOMESCO, JFH20 - Proctocolectomy with ileostomy)

## Discussion

### Main findings

This study aimed to validate IBD-related surgical procedure codes and the PPV, sensitivity and specificity of those codes in the NPR. We reviewed the charts of 262 randomly selected patients in Sweden of whom 57 (22%) underwent IBD surgery registered in the NPR between 1966 and 2014. Of these, 4 were excluded due to insufficient data on type of surgery in the patient charts to allow any reliable validation. For the remaining 53 patients, 158 codes were registered in the NPR. Of these 158, 155 (representing 60 different surgical procedure codes) were also present in the patient charts and validated using the charts as the gold standard for the validation. Our study showed a PPV of 96.8% for concordant codes (*n* = 153) registered in the NPR and a sensitivity for any of the validated codes (*n* = 155) of 94.5%.

### Comparison to other studies

Very few studies have validated the quality of data of surgical procedures codes in the NPR. Lagergren and Derogar found an overall PPV of 99.6% (*n* = 1358) for oesophageal cancer surgery [17]; Falkeborn et al., assessing gynaecological surgery, found PPVs ranging from 86 to 100% (*n* = 1338) depending on the type of surgery [21]; and in the most recent study, Tao et al. reported an overall PPV of 97.0% (*n* = 572) for obesity surgery codes [18]. Outside Scandinavia, Ma et al. reported PPVs from 80 to 100% (*n* = 113) for surgical resection procedure codes in patients with CD registered in the Calgary Health Zone discharge administrative database [5]. Our results are similar, although we validated a larger number of procedure codes compared with that and other studies. Analyses of subgroups show PPVs of 94.1% for abdominal codes, 100% for perianal codes and 98.1% for other IBD-related surgery codes. These findings correspond well with the overall PPV. Our PPV results for abdominal resection codes could be compared with those reported by Ma et al. of 87, 81 and 100% for partial excision of small intestine, partial excision of large intestine and total excision of large intestine, respectively [5]. The coverage of the NPR has changed over time. From 1997 and onwards, the NPR includes day surgery. In 1993, it became mandatory to register surgical procedure codes (such registration was however done to a considerable extent also before 1993). Our study included codes registered between 1966 and 2014. Falkeborn et al. validated codes for gynaecological surgery registered in 1965–1983 [21] and Lagergren and Derogar for oesophageal surgery in 1987–2005 [17]. The different periods of inclusion may limit comparisons with our study. Tao et al. included patients only during 2011, making direct comparisons difficult [18]. Sensitivity studies on the NPR are scarce. A sensitivity of 91% was found for any surgical procedure code during hospital admission of 962 patients in 1986 [22] and a sensitivity of over 97% for gynaecological procedure codes in 1965–1983 [21] was reported in another study. Both these studies were conducted before it became mandatory to register procedure codes in the NPR, whereas our study included codes both before and after that requirement. The higher sensitivity for gynaecological codes could be related to a smaller number of different codes in that study. We also included minor surgical interventions, such as perianal procedures, which are possibly less likely to be registered in the NPR because of the less complicated nature of the procedures. Our results show a sensitivity of 94.5% for IBD-related surgical procedure codes, which is consistent with studies of similar codes in the NPR. However, it is higher than the sensitivity of 79–86% reported by Ma et al. [5]. The classification system for procedure codes changed in 1997. When comparing procedure codes up until 1996 and 1997 or later, we found sensitivities of 90.6 and 98.7%. We speculate that the higher sensitivity 1997 and onwards could be related to the introduction in 1993 of mandatory registration of surgical procedures in the NPR. However, any differences over time should be interpreted with caution due to small numbers.

### Strengths and limitations

Retrospective review of patient charts should be the method of choice when validating surgical procedure codes in the NPR. This methodology has several strengths, including accurately determining the concordance between the charts and the NPR and a possibility to accurately categorise the types of error. Further strengths of our study include the random and nationwide population-based sampling of patients that reduces the risk of selection bias. Moreover, our study included surgical procedure codes registered between 1966 and 2014, allowing for assessment of the PPV and sensitivity of the NPR over time. The Swedish healthcare system, offering free access to equal care regardless of income and place of residency, provides high external validity as compared with similar healthcare systems such as those of the other Scandinavian countries. The results of this study give an estimate of the validity of surgical procedure codes for IBD related surgery in the NPR previously not known. The results allow future studies to accurately investigate the efficacy of surgery and surgical complications in various subgroups of IBD related surgical procedure codes in the NPR. The overlap of codes between IBD related surgery and other abdominal or perianal surgery lends the results external validity. This study has some limitations. Because reviewed charts were drawn from a sample of a previous validation study [16] that did not receive all requested charts, we cannot exclude that missing charts have biased our results. However, the patients included still represent a random nationwide sample and our 20% surgery rate (53/258) for the included patients can be compared to an expected lifetime risk of surgery of 20 to 50% for IBD-patients [2–8]. We do not expect the missing charts to be significantly different from the charts included. In addition, because the classification system for procedure codes changed in 1997, it cannot be excluded that the larger amount of procedure codes introduced after the change influenced the risk of misclassification. However, the notes in the charts served as the gold standard and therefore the risk of misclassification caused by the individual surgeon was minimal. The proportion of reviewed patient charts that included specific surgical notes was 71% (*n* = 110). Review of the remaining charts was based on other notes, which increases the risk of misclassification of these cases compared with cases confirmed using surgical notes. This limitation, however, was addressed by including only those procedures supported by other unambiguous notes that are equivalent to surgical notes. The manual review of the charts provides a robust reviewing process. Still, it could introduce misclassification by technical translation errors or by human error. The abstracted data were therefore reviewed twice by AF to minimise transfer errors. Furthermore, the surgical notes used for validation included detailed separate descriptions of the surgical procedures and techniques used, reducing the risk of misclassification. The review was done by a single reviewer. The chart reviewer was not blinded, which might have biased the assessment of the codes. The number of procedure codes used in IBD-related surgery is larger than the number of validated codes in this study. Nevertheless, we found and validated 60 different types of surgical procedure codes that covered the most frequently used procedures in IBD surgery (Additional file 1: Table S1). Although the validation was limited to patients with at least one IBD diagnosis in the NPR, the validity of the investigated procedure codes is likely to be generalisable to patients without IBD. The 95% CIs for presented accuracy measures was adjusted for clustering only on hospital level using a two-step bootstrap approach. There is to our knowledge no support for clustering also on lower hierarchical levels [19, 20]. The clustering was made in strict hierarchy with the exception of one patient who underwent surgery in two different hospitals. Finally, because only the admission date is usually listed in the NPR, we explored the actual date of surgery through patient charts. In studies examining outcomes after surgery we recommend that the difference between hospital admission date and the actual date of surgery (in this study median: 1 day, mean: 2.1 days) is taken into account.

## Conclusions

This nationwide study of 262 patients having at least one IBD diagnosis found a high positive predictive value and a high sensitivity and specificity of IBD-related surgical procedure codes registered in the NPR. Such a finding indicates that the NPR is a reliable data source for identifying patients that have undergone IBD-related surgery.

## Supplementary information

**Additional file 1: Table S1.** IBD-related surgical procedure codes included in the review process with frequency of validated codes; 2) **Table S2.** Classification and definitions of coding errors in the NPR with frequency Table S1 includes all surgical procedure codes included in the review process with frequency of validated codes. Table S2 shows the classification and definitions of coding errors used in the validation process together with frequency of identified errors.

## Data Availability

The datasets used and analysed during the current study are available from the corresponding author on reasonable request.

## Abbreviations

CD: Crohn’s disease
IBD: Inflammatory bowel disease
ICD: International classification of disease
NPR: National Patient Register
PIN: Personal identity number
PPV: Positive predictive value
UC: Ulcerative colitis
