# Supplementary material for: Validating surgical procedure codes for inflammatory bowel disease in the Swedish National Patient Register
Source: BMC Med Inform Decis Mak. 2019 Nov 11;19:217. doi: 10.1186/s12911-019-0948-z (PMC6849315; doi:10.1186/s12911-019-0948-z)
Supplement: Supplementary file 1 — Additional file 1: Table S1. IBD-related surgical procedure codes included in the review process with frequency of validated codes; 2) Table S2. Classification and definitions of coding errors in the NPR with frequency Table S1 includes all surgical procedure codes included in the review process with frequency of validated codes. Table S2 shows the classification and definitions of coding errors used in the validation process together with frequency of identified errors. [file 12911_2019_948_MOESM1_ESM.docx]

**Supplementary tables**

**Table S1: IBD-related surgical procedure codes included in the review process with frequency of validated codes**

| **Abdominal resection** | | **(n=)** |
| --- | --- | --- |
| **Resection of small bowel with/without formation of a stoma** | | |
| **4630*** | Resection of small bowel (with an end-to-end anastomosis ) | 12 |
| **4631** | Resection of small bowel (with other type of anastomosis) | 1 |
| JFB00** | Resection of small bowel | 7 |
| JFB01 | Laparoscopic resection of small bowel |  |
| **0053** | Formation of a jejunostomy (when performed in conjunction with resectional procedures) |  |
| **0054** | Formation of an ileostomy (when performed in conjunction with resectional procedures) |  |
| **4661** | Exteriorisation of bowel with enterotomy |  |
| JFF10 | Formation of a loopileostomy |  |
| JFF11 | Laparoscopic formation of a loopileostomy |  |
| JFF96 | Other formation of stoma on small bowel or colon |  |
| JFF97 | Other laparoscopic formation of stoma on small bowel or colon |  |
| **Ileocaecal, ileocolic resection or right-sided hemicolectomy with/without formation of a stoma** | | |
| **4641** | Right-sided hemicolectomy | 4 |
| **4642** | Ileocaecal resection | 11 |
| **4648** | Ileocolic reresection | 1 |
| JFB20 | Ileocaecal resection | 11 |
| JFB21 | Laparoscopic ileocaecal resection |  |
| JFB30 | Right-sided hemicolectomy | 1 |
| JFB31 | Laparoscopic right-sided hemicolectomy |  |
| JFB33 | Other resection of small bowel and colon, including reresection | 1 |
| JFB34 | Laparoscopic other resection of small bowel and colon, including reresection |  |
| **0053** | Formation of a jejunostomy (in conjunction with resectional procedures) |  |
| **0054** | Formation of an ileostomy (when performed in conjunction with resectional procedures) |  |
| **4661** | Exteriorisation of bowel with enterotomy |  |
| JFF10 | Formation of a loopileostomy |  |
| JFF11 | Laparoscopic formation of a loopileostomy |  |
| JFF96 | Other formation of stoma on small bowel or colon |  |
| JFF97 | Other laparoscopic formation of stoma on small bowel or colon |  |
| **Resection: rectum, sigmoid or transverse colon, left-sided hemicolectomy with/without stoma formation** | | |
| **4640** | Left-sided hemicolectomy |  |
| **4643** | Resection of transverse colon |  |
| **4644** | Resection of the sigmoid colon |  |
| **4649** | Other form of resection of colon |  |
| **4713** | Formation of a stoma on the sigmoid colon and closure of the distal bowel end |  |
| **4821** | Resection of rectum |  |
| **4828** | Operations similar to resection of rectum | 1 |
| JFB40 | Resection of the transverse colon | 1 |
| JFB41 | Laparoscopic resection of the transverse colon |  |
| JFB43 | Left-sided hemicolectomy |  |
| JFB44 | Laparoscopic left-sided hemicolectomy |  |
| JFB46 | Resection of the sigmoid colon |  |
| JFB47 | Laparoscopic resection of the sigmoid colon |  |
| JFB50 | Other resection of colon | 1 |
| JFB51 | Laparoscopic other resection of colon |  |
| JFB60 | Resection of sigmoid colon, closure of rectum and formation of a colostomy |  |
| JFB61 | Laparoscopic resection of sigmoid colon and formation of a colostomy |  |
| JFB63 | Other resection of colon with closure of distal bowel end and formation of a colostomy |  |
| JFB64 | Laparoscopic other resection of colon with closure of distal bowel end and formation of a colostomy |  |
| JGB00 | Resection of rectum |  |
| JGB01 | Laparoscopic resection of rectum |  |
| JGB10 | Hartmann's resection |  |
| JGB11 | Laparoscopic Hartmann's resection |  |
| **4710** | Formation of a stoma on the sigmoid colon |  |
| **4711** | Formation of a stoma on the transverse colon |  |
| JFF10 | Formation of a loopileostomy |  |
| JFF11 | Laparoscopic formation of a loopileostomy |  |
| JFF23 | Formation of a stoma on the transverse colon |  |
| JFF24 | Laparoscopic of a stoma on the transverse colon |  |
| JFF26 | Formation of a stoma on the sigmoid colon | 1 |
| JFF27 | Laparoscopic formation of a stoma on the sigmoid colon |  |
| JFF30 | Other formation of colostomy |  |
| JFF31 | Laparoscopic other formation of colostomy |  |
| JFF96 | Other formation of stoma on small bowel or colon |  |
| JFF97 | Other laparoscopic formation of stoma on small bowel or colon |  |
| **Colectomies with or without formation of a stoma** | | |
| **4650** | Colectomy with IRA |  |
| **4651** | Colectomy and formation of an ileostomy | 3 |
| **4652** | Proctocolectomy and formation of an ileostomy |  |
| JFH00 | Colectomy with ileorectal anastomosis | 1 |
| JFH01 | Laparoscopic colectomy with ileorectal anastomosis |  |
| JFH10 | Colectomy with ileostomy | 5 |
| JFH11 | Laparoscopic colectomy with ileostomy | 1 |
| JFH20 | Proctocolectomy with ileostomy |  |
| JFH96 | Other colectomy |  |
| **0054** | Formation of an ileostomy (when performed in conjunction with resectional procedures) |  |
| **4661** | Exteriorisation of bowel with enterotomy |  |
| JFF10 | Formation of a loopileostomy |  |
| JFF11 | Laparoscopic formation of a loopileostomy |  |
| JFF96 | Other formation of stoma on small bowel or colon |  |
| JFF97 | Other laparoscopic formation of stoma on small bowel or colon |  |
| **Proctectomy** | | |
| **4820** | Abdominoperineal rectal resection | 2 |
| JGB30 | Abdominoperineal rectal resection |  |
| JGB31 | Laparoscopic abdominoperineal rectal resection |  |
| **Perianal procedures** | | |
| JHA00 | Anal or perianal incision | 3 |
| JHA10 | Biopsy of anal canal | 1 |
| JHD20 | Incision of fistula-in-ano | 3 |
| JHD30 | Incomplete incision of fistula-in-ano | 7 |
| JHD33 | Completing incision of fistula-in-ano | 1 |
| JHW96 | Other operation on anus or perianal tissue | 4 |
| **4899** | Other operation on rectum or perirectal tissue |  |
| **4922** | Incision of fistula-in-ano | 2 |
| **4970** | Anal or perianal incision | 3 |
| **4999** | Other operation on anus or perianal tissue | 12 |
| **Other surgery – IBD** | | |
| **Other IBD-related abdominal surgery including formation of a stoma as the only procedure** | | |
| **4010** | Explorative laparotomy | 6 |
| **4030** | Laparotomy and drainage |  |
| **4031** | Incision and drainage | 2 |
| **4611** | Laparatomy and closure of a fistula to the bowel wall |  |
| **4653** | Proctocolectomy and formation of a continent ileostomy |  |
| **4654** | Proctocolectomy and ileoanal anastomosis |  |
| **4660** | Exteriorisation of bowel | 1 |
| **4661** | Exteriorisation of bowel with enterotomy |  |
| **4664** | Closure of ileostomy | 1 |
| **4700** | Formation of an ileostomy as the only procedure | 1 |
| **4710** | Formation of a stoma on the sigmoid colon |  |
| **4730** | Duodenojejunostomy |  |
| **4731** | Enteroanastomosis |  |
| **4732** | Ileocolostomy |  |
| **4733** | Colo-colostomy |  |
| **4734** | Ileotransversostomy | 1 |
| **4735** | Ileosigmoidostomy |  |
| **4739** | Operations similar to different enteric/ colonic anastomoses without resection | 2 |
| **4740** | Suture of the small bowel wall |  |
| **4741** | Suture of the colonic wall |  |
| **4770** | Freeing of adhesions in the peritoneal cavity | 5 |
| **4771** | Enterolysis |  |
| **4790** | Revision of a stoma without laparotomy | 1 |
| **4791** | Laparatomy and revision of a stoma | 1 |
| **4792** | Laparatomy and formation of a new ileostomy |  |
| **4793** | Conversion of a conventional ileostomy to a continent ileostomy | 1 |
| **4794** | Revision of a continent ileostomy |  |
| **4795** | Excision of ileal pouch anal anastomosis and creation of a conventional ileostomy |  |
| **4796** | Transposition of ileostomy |  |
| **4797** | Transposition of colostomy |  |
| **4798** | Operations similar to transposition of stoma | 1 |
| **4822** | Extirpation of rectum and formation of a continent ileostomy |  |
| **4823** | Extirpation of rectum and creation of an ileal pouch anal anastomosis |  |
| JAA00 | Incision of abdominal wall (validated together with a concurrent IBD-related code) | 1 |
| JAH00 | Laparotomy | 3 |
| JAH01 | Laparoscopy |  |
| JAP00 | Freeing of adhesions in the peritoneal cavity | 1 |
| JFA60 | Enteric strictureplasty |  |
| JFA63 | Colonic strictureplasty |  |
| JFA70 | Suture of the small intestine (closure of perforation or laceration) | 1 |
| JFA71 | Laparoscopic suture of the small bowel wall |  |
| JFA76 | Closure of a fistula to the small bowel | 1 |
| JFA80 | Suture of the colonic wall |  |
| JFA81 | Laparoscopic suture of the colonic wall |  |
| JFA86 | Closure of a colonic fistula | 1 |
| JFA96 | Other local operation on intestine | 1 |
| JFB96 | Other resection of small bowel or colon | 3 |
| JFB97 | Laparoscopic other resection of small bowel or colon |  |
| JFC00 | Enteroanastomosis |  |
| JFC01 | Laparoscopic enteroanastomosis |  |
| JFC10 | Ileotransversostomy | 1 |
| JFC11 | Laparoscopic ileotransversostomy |  |
| JFC20 | Other entero-colostomy |  |
| JFC21 | Other laparoscopic entero-colostomy |  |
| JFC30 | Colo-colostomy |  |
| JFC31 | Laparoscopic colo-colostomy |  |
| JFC40 | Ileorectostomy | 1 |
| JFC50 | Colorectal anastomosis |  |
| JFC51 | Laparoscopic colorectal anastomosis |  |
| JFF13 | Formation of a enterostoma | 2 |
| JFG00 | Closure of loop enterostomy without resection | 2 |
| JFG10 | Closure of loop colostomy without resection | 1 |
| JFG20 | Closure of enterostomy with resection of exteriorised loop | 2 |
| JFG26 | Closure of terminal enterostomy with anastomosis to colon | 1 |
| JFG40 | Revision of enterostomy or colostomy without laparotomy |  |
| JFG50 | Revision of enterostomy or colostomy with laparotomy |  |
| JFG53 | Revision of ileal pouch anal anastomosis |  |
| JFG56 | Revision of colonic pouch |  |
| JFG60 | Conversion of a conventional ileostomy to a continent ileostomy |  |
| JFG70 | Conversion of a continent ileostomy to a conventional ileostomy |  |
| JFG73 | Excision of ileal pouch anal anastomosis |  |
| JFG76 | Excision of colonic pouch with colorectal or coloanal anastomosis |  |
| JFG80 | Excision of ileal pouch anal anastomosis and creation of continent ileostomy |  |
| JFG83 | Redo of colonic pouch anal anastomosis |  |
| JFG86 | Redo of ileal pouch anal anastomosis |  |
| JFG96 | Other operation of stoma or pouch |  |
| JFH30 | Colectomy, rectal mucosectomy, ileoanal anastomosis without stoma |  |
| JFH33 | Colectomy, rectal mucosectomy, ileoanal anastomosis with formation of an ileostomy |  |
| JFH40 | Proctocolectomy with formation of a continent ileostomy |  |
| JFK00 | Division of adhesive band in intestinal obstruction | 1 |
| JFK10 | Freeing of adhesions in intestinal obstruction | 3 |
| JFK20 | Freeing of adhesions and plication of small intestine |  |
| JFL96 | Other operation on adhesions in intestinal obstruction | 1 |
| JFW96 | Other operation of small bowel or colon |  |
| JFW97 | Other laparoscopic operation on small bowel or colon |  |
| JGB50 | Ileal pouch anal anastomosis with mucosectomy |  |
| JGB60 | Ileal pouch anal anastomosis without mucosectomy |  |
| JGB96 | Other rectal resection or extirpation | 1 |
| JGB97 | Other laparoscopic rectal resection or extirpation |  |
| JWF00 | Reoperation for insufficiency of anastomosis or suture in gastroenterological surgery | 2 |
| KCH30 | Closure of vesicointestinal fistula | 1 |
| **Total (n=)** | | 155 |

*IBD = inflammatory bowel disease*

** Procedure codes according to the Swedish classification system between 1964 and 1996 in bold letters*

*** Procedure codes according to the NOMESCO Classification 1997 onwards*

**Table S2: Classification and definitions of coding errors in the NPR with frequency**

| **Type of error** | **Definition** | **(n=)** |
| --- | --- | --- |
| Absence of code | Procedure code in the chart not in the NPR | 9 |
| Transfer error | Procedure code in the chart not the same in the NPR | 2 |
| Descriptive error | Procedure code in the chart not supported by surgery notes or other notes | 0 |

*NPR = national patient register*

**Supplementary table legends**

**Table S1: IBD-related surgical procedure codes included in the review process with frequency of validated codes**

*IBD = inflammatory bowel disease*

** Procedure codes according to the Swedish classification system between 1964 and 1996 in bold letters*

*** Procedure codes according to the NOMESCO Classification 1997 onwards*

**Table S2: Classification and definitions of coding errors in the NPR with frequency**

*NPR = national patient register*
